# Inhibition of Glycolysis Impairs Retinoic Acid-Inducible Gene I–Mediated Antiviral Responses in Primary Human Dendritic Cells

**DOI:** 10.3389/fcimb.2022.910864

**Published:** 2022-07-18

**Authors:** Alessandra Zevini, Enrico Palermo, Daniele Di Carlo, Magdalini Alexandridi, Serena Rinaldo, Alessio Paone, Francesca Cutruzzola, Marilena P. Etna, Eliana M. Coccia, David Olagnier, John Hiscott

**Affiliations:** ^1^ Pasteur Laboratories, Istituto Pasteur Italia - Fondazione Cenci Bolognetti, Rome, Italy; ^2^ Department of Biochemical Sciences “A. Rossi Fanelli”, Sapienza University of Rome, Rome, Italy; ^3^ Department of Infectious Diseases, Istituto Superiore Sanità, Rome, Italy; ^4^ Department of Biomedicine, Aarhus University, Aarhus, Denmark

**Keywords:** innate immunity, immunometabolism, viral infection, glycolysis, RIG-I, moDC

## Abstract

Dendritic cells (DCs) are important mediators of the induction and regulation of adaptive immune responses following microbial infection and inflammation. Sensing environmental danger signals including viruses, microbial products, or inflammatory stimuli by DCs leads to the rapid transition from a resting state to an activated mature state. DC maturation involves enhanced capturing and processing of antigens for presentation by major histocompatibility complex (MHC) class I and class II, upregulation of chemokines and their receptors, cytokines and costimulatory molecules, and migration to lymphoid tissues where they prime naive T cells. Orchestrating a cellular response to environmental threats requires a high bioenergetic cost that accompanies the metabolic reprogramming of DCs during activation. We previously demonstrated that DCs undergo a striking functional transition after stimulation of the retinoic acid-inducible gene I (RIG-I) pathway with a synthetic 5′ triphosphate containing RNA (termed M8), consisting of the upregulation of interferon (IFN)–stimulated antiviral genes, increased DC phagocytosis, activation of a proinflammatory phenotype, and induction of markers associated with immunogenic cell death. In the present study, we set out to determine the metabolic changes associated with RIG-I stimulation by M8. The rate of glycolysis in primary human DCs was increased in response to RIG-I activation, and glycolytic reprogramming was an essential requirement for DC activation. Pharmacological inhibition of glycolysis in monocyte-derived dendritic cells (MoDCs) impaired type I IFN induction and signaling by disrupting the TBK1-IRF3-STAT1 axis, thereby countering the antiviral activity induced by M8. Functionally, the impaired IFN response resulted in enhanced viral replication of dengue, coronavirus 229E, and Coxsackie B5.

**Graphical Abstract:**
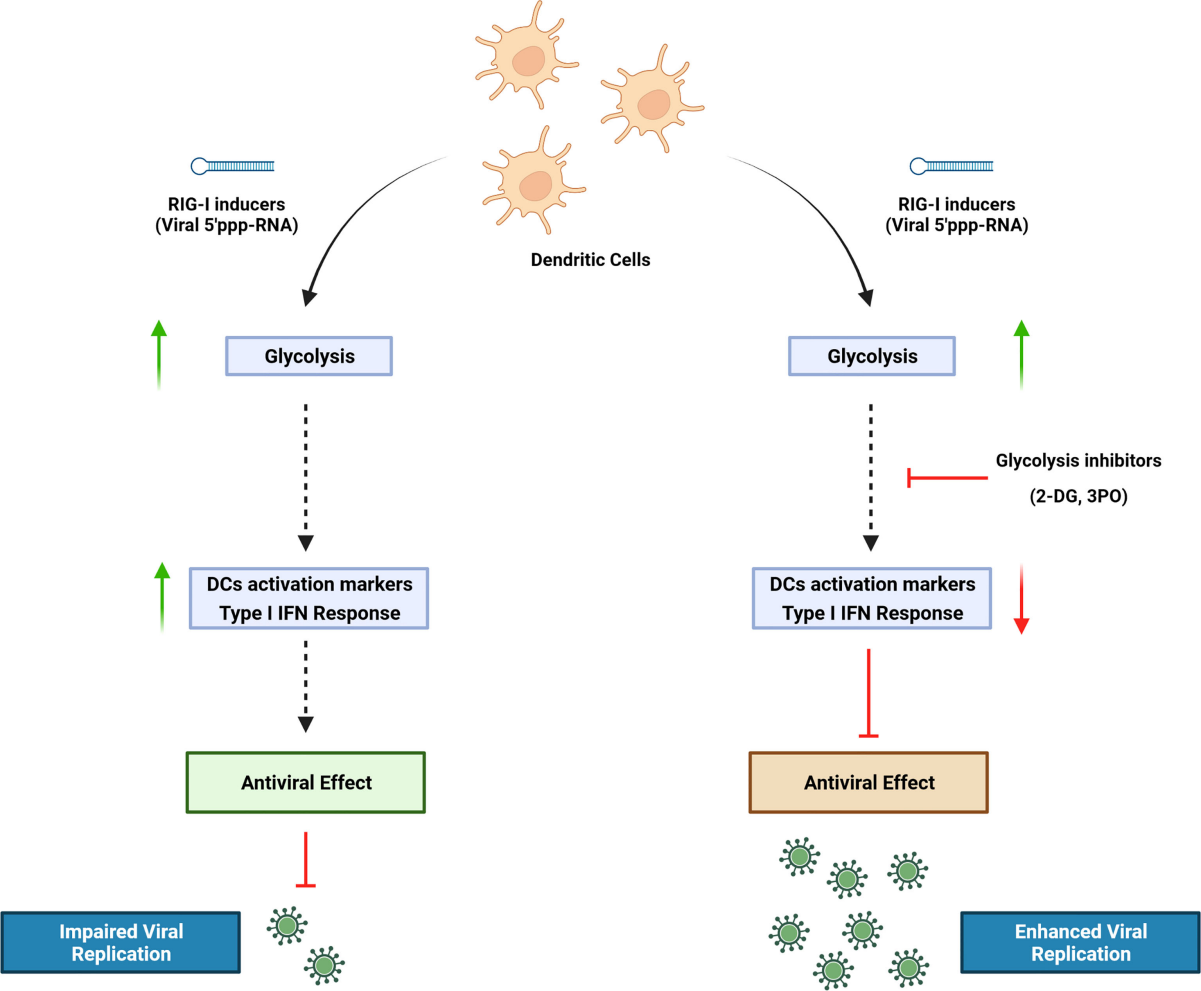


## Introduction

Dendritic cells (DCs) play an essential role in the induction and regulation of immune response during microbial infection and inflammation. The expression of a wide repertoire of pattern recognition receptors (PRRs), including retinoic acid-inducible gene I (RIG-I), enables them to sense danger signals, as microbial-derived products or inflammatory stimuli, and to rapidly switch from a resting state to an activated, matured state. Orchestrating a response to environmental threats is a process that carries a high bioenergetic cost. A profound metabolic reprogramming occurs in DCs undergoing activation (Ganeshan and Chawla, 2014). Nutrients are taken up at a higher rate to quickly generate the energy required to sustain the new cellular programs in activated immune cells; moreover, the intermediates produced during metabolism of nutrients provide the building blocks for the synthesis of macromolecules such as RNA, DNA, proteins, and membranes, thus supporting the development of demanding new biosynthetic activities of DCs (Ganeshan and Chawla, 2014; Cabeza-Cabrerizo et al., 2021).

Understanding how immune cells reprogram their metabolism in response to activating stimuli can have significant therapeutic implications. For this reason, considerable research has focused on characterizing the metabolic transition driven by Toll-like receptors (TLRs) in myeloid cells (Krawczyk et al., 2010; Everts et al., 2014; Mills et al., 2016; Lee et al., 2019): Several studies demonstrated that DCs—as well as monocytes and macrophages—exposed to Lipopolysaccharide (LPS) switch to a highly glycolytic profile, and that glucose restriction inhibits their activation. In addition, stimulation of RIG-I pathway, a key sensor of virus infection, was shown to induce metabolic reprogramming by activating glycolysis, which is necessary for interferon (IFN) production in monocyte-derived dendritic cells (MoDCs) (Fekete et al., 2018). However, the role of glycolysis in RIG-I–mediated response during viral infections has not been defined.

RIG-I is a cytosolic PRR for short 5’ di- and triphosphate double-stranded RNA and represents a critical component in the activation of innate antiviral response (Hornung et al., 2006). Upon recognition of viral RNA, RIG-I undergoes a conformational transition and associates with the mitochondrial antiviral signaling protein (MAVS) adaptor, leading in turn to the activation of the tank-binding kinase 1 (TBK1) and the IκB kinase (IKK) complex (Kawai et al., 2005). These events culminate with the phosphorylation and activation of interferon regulatory factor 3 (IRF3) and nuclear factor κB (NF-κB), respectively, resulting in the induction of the IFN antiviral and inflammatory responses (Goulet et al., 2013; Zevini et al., 2017). As a mechanism of protection against uncontrolled viral spreading, RIG-I signaling can also lead to immunogenic death of infected cells, indicating a potential anti-cancer activity of RIG-I agonists. Recently, these compounds showed a strong killing activity in different tumor types, leading to the activation of both innate and adaptive immune responses against tumors in mouse models (Poeck et al., 2008; Besch et al., 2009; Ellermeier et al., 2013; Glas et al., 2013; Duewell et al., 2014).

In this study, we used a RIG-I agonist (termed M8) derived from vesicular stomatitis virus (VSV) RNA. Compared with other agonists, M8 holds a stronger ability to stimulate antiviral immunity (Chiang et al., 2015) and blocks viral infections *in vitro* and *in vivo* by activating a broader and intense innate immune response (Beljanski et al., 2015). DCs undergo a striking functional transition after stimulation of the RIG-I pathway with M8, involving the pronounced upregulation of antiviral genes, an increased phagocytic potential, activation of a marked proinflammatory phenotype, and the induction of markers associated with immunogenic cell death (Chiang et al., 2015; Castiello et al., 2019). In the present study, we demonstrate that DC increased their glycolytic rate in response to M8, and that glycolysis was an essential requirement for DC activation; pharmacological or genetic inhibition of M8 activity resulted in the failure of DC maturation and highly diminished antiviral defense. Finally, impairment of the M8 antiviral response using pharmacological inhibition of glycolysis increased dengue, human coronavirus 229E and Coxsackie B5 infection by promoting viral replication.

## Results

**Figure 1:**
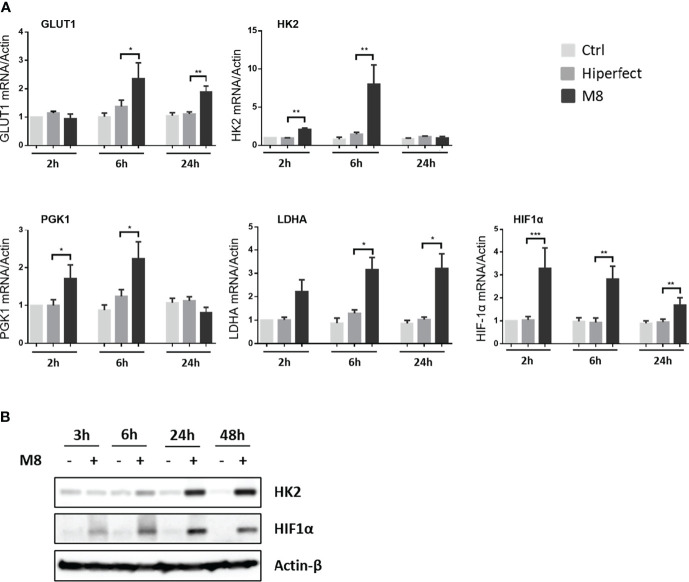
Analysis of metabolic enzyme expressions during monocyte-derived dendritic cells (MoDCs) maturation by M8. **(A)** CD14+ monocytes isolated from freshly collected blood of healthy donors were differentiated in the presence of GM-CSF (Granulocyte-macrophage colony-stimulating factor) and IL-4 for 5 days and stimulated with 50 ng/ml of M8 for 2–24h. RNA was extracted and used for RT-PCR analyses of glycolytic molecules (GLUT1, HK2, PGK1, LDHA, and HIF1α). RNA expression was normalized on β-actin, and results represent fold changes compared with CTRL, set as 1. Data are representative of four independent experiments (*n* = 4). **(B)** Immunoblot analyses of HK2 and HIF-1α in MoDCs treated or not for the indicated hours by M8 (50 ng/ml), protein amount was normalized on GAPDH protein expression.

*RIG-I agonist M8 triggers changes in the metabolic gene expression profile of DCs.* To determine the effect of the RIG-I agonist M8 on the MoDCs metabolic profile, we performed a quantitative real-time polymerase chain reaction (PCR) to analyze the transcriptional regulation of some key glycolytic enzymes. mRNA expression of hexokinase 2 (HK2), the first enzyme in the glycolytic pathway; phosphoglycerate kinase (PGK); lactate dehydrogenase A (LDHA); hypoxia-inducible factor 1α (HIF-1α), a principal regulator of metabolism, controlling most genes encoding glycolytic enzymes in mammalian cells (Semenza et al., 1994; Blouin et al., 2004); and the glucose transporter Glut1 (**Figure 1A**) were upregulated in the presence of M8, whereas glyceraldehyde-3-phosphate dehydrogenase (GAPDH), a gene with constitutive expression, was not affected by M8 (data not shown). M8 transcriptional regulation was sufficient to affect protein levels, as MoDCs displayed increasing levels of HK2 and HIF-1α proteins over M8 treatment (**Figure 1B**). Collectively, these data suggest that RIG-I signaling activation by M8 impacted MoDCs metabolism—at least in part—by HIF-1α.

**Figure 2:**
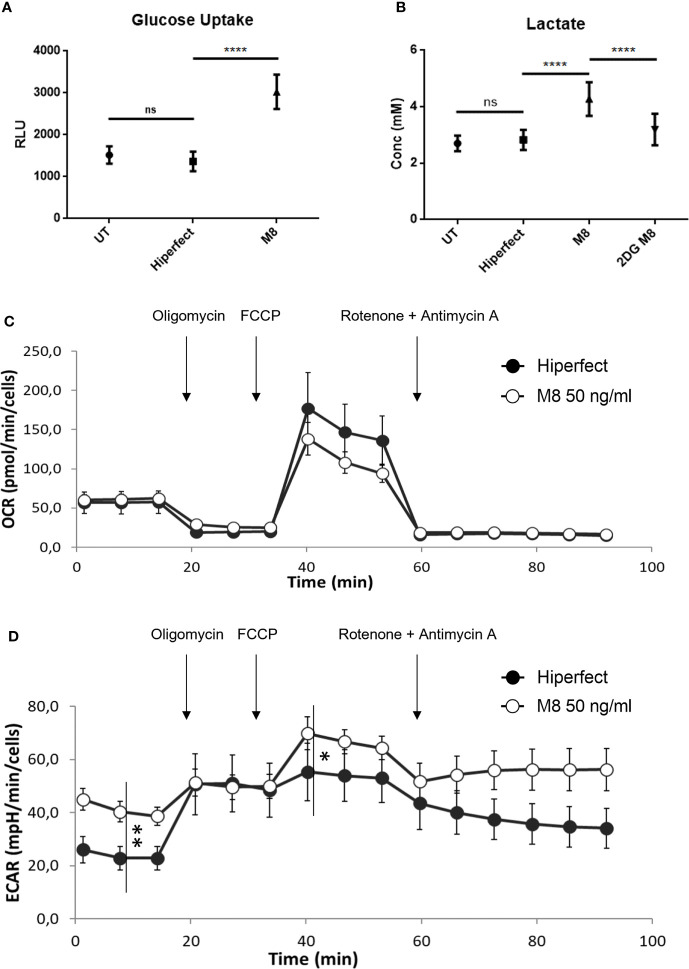
M8 modulates glucose metabolism and bioenergetic profile in monocyte-derived dendritic cells (MoDCs). **(A)** Eighteen hours after M8 treatment, MoDCs were incubated with 2-deoxyglucose (2-DG) for 20 min, washed, detached, and their 2-DG uptake capacity was measured by a colorimetric assay, as a measure of glucose uptake. Data are means ± SD of duplicates, obtained from three independent experiments. ****P < 0.0001. **(B)** MoDCs were stimulated with 50 ng/ml of M8 ± 6 mM 2-DG for 18h and lactate concentration in the medium was measured *via* a bioluminescent assay. Data are means ± SD of duplicates, obtained from six independent experiments. ****P < 0.001. **(C)** The mitochondrial respiration of MoDCs obtained by means of OCR by seahorse experiments and **(D)** the corresponding glycolysis obtained by means of ECAR after M8 treatment (open circles) compared to transfection control (black circles). Arrows indicate the addiction of drugs used to specifically target the mitochondrial function (i.e., oligomycin, FCCP, rotenone + antimycin, in order of addiction. *P < 0.01; **P < 0.0001. In the figure a representative experiment; values reported in the plot are the means of least 10 replicates ± SD.

M8 induces a glycolytic switch in MoDCs. Increased glycolysis requires higher availability of intracellular glucose; consistent with the upregulated expression of GLUT1, M8-stimulated MoDCs showed increased glucose uptake, compared with non-stimulated MoDCs, or MoDCs transfected only with HiPerFect (**Figure 2A**). To further determine whether the changes observed in gene expression had a significant functional impact, we measured lactate production, a metabolic end point of aerobic glycolysis, in the presence of M8: MoDCs showed higher amount of lactate in the supernatant, indicating an increased glycolytic activity; in support of this observation, we also observed that pharmacological inhibition of HK2 by the 2-deoxyglucose (2-DG) inhibitor, completely abrogated this effect (Grossbard and Schimke, 1966) (**Figure 2B**).

To better characterize the effect of M8 treatment in shaping metabolism, the energetic profile of MoDCs was assessed using the Seahorse extracellular flux analyzer XFe96. This platform allows the determination of the oxygen consumption rate (OCR) under both basal and stressed conditions (**Figure 2C**), which is indicative of the mitochondrial function; at the same time, the extracellular acidification rate (ECAR) was also measured as an indirect quantification of the glycolytic process (**Figure 2D**). As shown in **Figure 2**, while M8-induced OCR levels do not change, the corresponding basal ECAR increased significantly, thus indicating that glycolysis served as the main energy source to support DC activation (Everts et al., 2012). Interestingly, ECAR increase is also observed upon addition of respiratory stressors such as oligomycin and carbonyl cyanide *p*trifluoromethoxyphenylhydrazone (FCCP) (see Methods for details). Thus, M8 treatment mainly boosted the glycolytic profile both under basal and stressed conditions, suggesting that (i) the large part of increased need of ATP production was sustained by an increased glycolytic process and (ii) this higher glycolysis is far from its maximal potential (as observed upon stressors addition), thus confirming a deep metabolic re-programming toward glycolytic profile rather than a simple speed up of the ongoing process.

### Inhibition of Glycolysis Impairs M8-Dependent MoDCs Maturation

**Figure 3:**
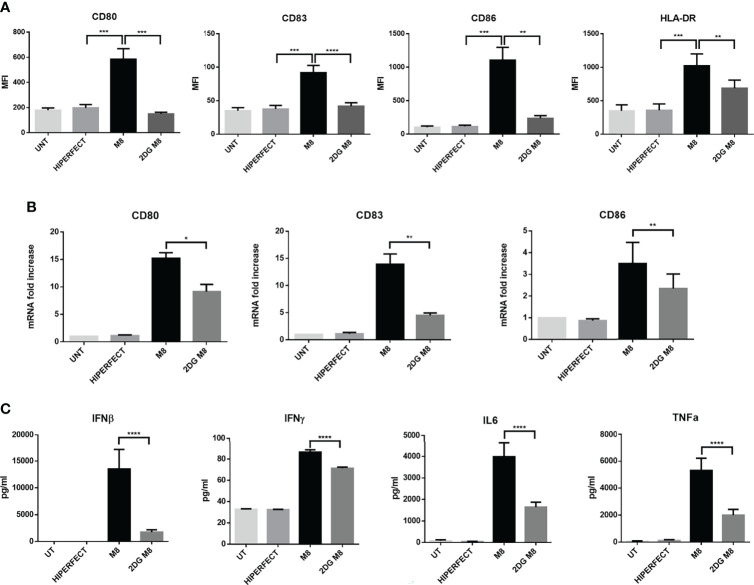
M8-induced upregulation of costimulatory molecules on monocyte-derived dendritic cells (MoDCs) requires glycolysis. **(A)** MoDCs were either treated or left untreated with 6 mM 2-deoxyglucose (2-DG) 1h prior exposition to 50 ng/ml of M8, and expression of MHC II, CD80, CD83, and CD86 on MoDCs were analyzed by flow cytometry after 18h. Histograms show the mean fluorescence intensity of the expression of the indicated molecule. Data are representative of six independent experiments. **(B)** MoDCs were treated as in **(A)**, RNA was isolated 24h after M8 treatment, and the expression of the indicated costimulatory mRNAs measured by RT-PCR. **(C)** Analysis by Magnetic Luminex assay of the concentration (pg/ml) of proinflammatory cytokines IL-6, TNF-α, IFN-γ, and IFN-β in supernatants of MoDCs stimulated for 24h with M8 (50 ng/ml) with or without 2-DG (6 mM) pretreatment. Statistical significance was defined as follows: *P < 0.05, **P < 0.01, ***P < 0.001, and ****P < 0.0001.

To investigate whether the early increase in glycolysis was important for M8-induced DC activation, we measured the level of costimulatory molecules CD80, CD83, and CD86, as well as of MHC-II on the surface of resting MoDCs, M8-treated MoDCs, and M8-treated MoDCs together with the glycolytic inhibitor 2-DG. M8-induced RIG-I activation resulted in enhanced expression of surface markers characteristic of functional maturation of MoDCs. When administered prior to M8, 2-DG decreased the expression of the three co-stimulatory molecules (CD80, -83, and -86) and MHC-II at 24h (**Figure 3A**). In addition, M8-induced expression of CD80, CD83, and CD86 mRNA was also significantly inhibited by 2-DG (**Figure 3B**), indicating that glycolysis sustains these markers of DC activation and maturation at the transcriptional level.

To characterize how glycolysis affects the cytokine secretion profile of MoDCs induced by M8, MoDCs were exposed to M8 in the absence or presence of 2-DG; at 24h, supernatants were harvested and analyzed by a multiplex enzyme-linked immunosorbent assay. MoDCs matured in the presence of M8 showed a higher capacity to produce proinflammatory cytokines, since interferon-β (IFN-β), interferon-γ (IFN-γ), interleukin-6 (IL-6), and tumor necrosis factor–α (TNF-α) were increased in the supernatants of MoDCs exposed to M8, whereas 2-DG pre-treatment reduced the amounts of these proinflammatory cytokines by 7.7-, 1.2-, 2.4-, and 2.6-fold, respectively (**Figure 3C**). Taken together, these data indicate that M8-matured human MoDCs require glycolysis to control their functional responses, including upregulation of costimulatory molecules and cytokine production.

### Glycolysis is Required for the Activation of RIG-I Signaling Pathway

**Figure 4:**
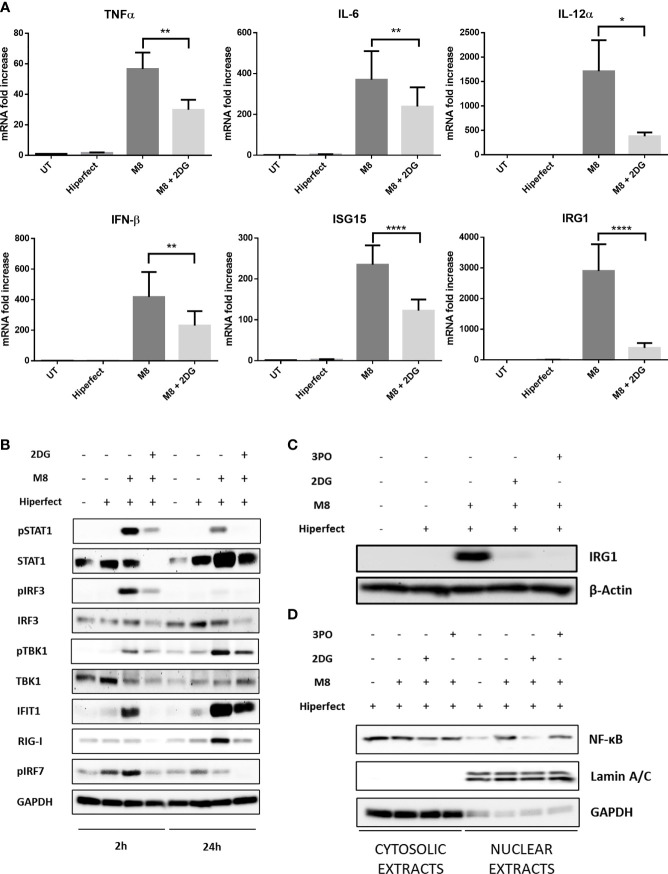
M8 requires active glycolysis to trigger type I interferon (IFN) and nuclear factor κB (NF-κB) responses in monocyte-derived dendritic cells (MoDCs). **(A)** Gene expression levels of IFN-β, ISG15, IRG1, IL-12, IL-6, and TNFα in MoDCs were quantified by qPCR after 1h treatment with or without 2-deoxyglucose (2-DG) (6 mM) and 6h treatment with M8 (50 ng/ml). Results are normalized to the TBP gene and expressed as fold of increase relative to control (UT), set as 1. Data represent means ± SEM from five independent experiments. **(B)** Immunoblot analysis of key components of type-I IFN signaling in MoDCs treated for the indicated hours by M8 alone (50 ng/ml) or pretreated by 2-DG (6 mM) for 1h, normalized on GAPDH expression. Culture medium and HiPerFect alone were used as controls. **(C)** Immunoblot analysis of IRG1 expression in MoDCs treated for 6h by M8 alone (50 ng/ml) or pre-treated by 2-DG (6 mM) or 3-PO (25 µM) for 1h, normalized on β-actin expression. Culture medium and HiPerFect alone were used as controls. **(D)** MoDCs were pre-treated or not with 2-DG (6 mM) or 3-PO (25 µM) for 1h, treated with M8 for 6h and subjected to subcellular fractionation. The levels of endogenous NF-κB in the nuclear and cytoplasmic fractions were determined by immunoblotting with anti–NF-κB p65 antibodies. The relative purity of the nuclear and cytoplasmic fractions was confirmed by sequential probing for the nuclear marker lamin A/C and the cytoplasmic marker GAPDH.

M8 stimulation induced significant alterations in the levels of immune-related transcripts; in particular, the proinflammatory genes TNF-α, IL-6, and IL-12α, as well as IFN-β increase several hundred fold in M8-treated MoDCs, compared with unstimulated MoDCs (**Figure 4A**); a similar pattern was observed for the IFN-stimulated gene 15 (ISG15), encoding for a ubiquitin-like intracellular protein, whose conjugation to various proteins (ISGylation) contributes to antiviral and antimycobacterial immunity (Bogunovic et al., 2012), and for immune-responsive gene 1 (IRG1), encoding the enzyme that catalyzes itaconate production and is involved in the regulation of inflammatory innate responses (Michelucci et al., 2013). Consistent with the results shown in **Figure 3**, inhibition of glycolysis also compromised the capacity of MoDCs to induce an antiviral transcriptional program in response to M8 stimulation; as shown in **Figure 4A**, 2-DG blocked the upregulation of IFN-β, the IFN-regulated genes ISG15 and IRG1, and the IL-6, IL-12α, and TNF-α transcripts. Thus, the burst of aerobic glycolysis early after stimulation with RIG-I agonist was required for activation of the antiviral and inflammatory transcriptional program in DCs.

Mechanistically, RIG-I drives DC activation *via* a dual signaling cascade by triggering the TBK1-IRF3 axis that leads to type I IFNs and ISGs expression and, in parallel, the NF-κB pathway. Since we observed an increase in ECAR within 2h of M8 stimulation, we sought to determine whether this increase in glycolysis was needed for the initial steps of RIG-I pathway induction. MoDCs stimulation with M8 led to the rapid phosphorylation of TBK1 and IRF3, necessary for the IFN-β–mediated STAT1 phosphorylation and IRG1 upregulation (**Figures 4B, C**). Moreover, M8 also rapidly activated the NF-κB response, as indicated by the nuclear translocation of NF-κB p65 (**Figure 4D**). Inhibition of glycolysis significantly blunted RIG-I signaling, as the combination of M8 + 2-DG showed significant impairment in the phosphorylation of TBK1, IRF3, and STAT1, reduced the levels of IRG1 protein expression and abrogated activation of NF-κB. A similar result was obtained in the presence of the 6-phosphofructo-2-kinase (PFK-2) inhibitor 3-PO (Emini Veseli et al., 2020) that also diminished the levels of IRG1 protein expression and the RIG-I response (**Figures 4C, D**).

### Glycolytic Inhibitors Restrict M8 Antiviral Potential

**Figure 5:**
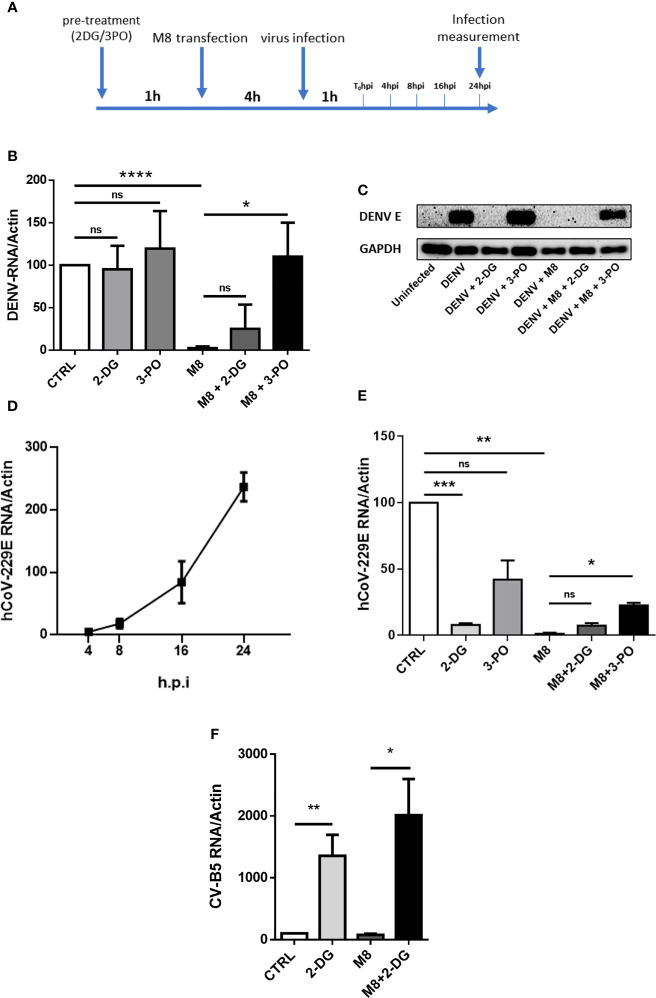
M8 requires glycolysis to exert its antiviral effect in monocyte-derived dendritic cells (MoDCs). **(A)** Schematic timeline representation of hCoV-229E and dengue virus (DENV) infection of human primary MoDCs. Previous stimuli were not removed when a new factor was added to cells. **(B)** quantitative polymerase chain reaction (qPCR) analysis of DENV RNA expression: MoDCs were treated with 2-deoxyglucose (2-DG) (7mM) or 3-PO (25 µM) for 1h and then transfected with M8 (0.5 ng/ml). After 4h cells were infected with DENV (MOI 5) for additional 24h. Cells were then harvested and expression of viral RNA was assessed by qPCR. DENV RNA expression was normalized on β-actin and results represent fold changes compared with CTRL (DENV alone), set as 100. **(C)** Immunoblot analysis representative of DENV E Protein expression in different samples. MoDCs were treated as in **(B)** then protein expression was analyzed by Western blot and viral protein expression was normalized on endogenous GAPDH. **(D)** qPCR analysis of hCoV-229E RNA expression. In order to assess viral replication, MoDCs were infected with human coronavirus 229E (MOI 1), cells were harvested at the indicated time points and viral RNA levels were measured by qPCR and normalized on β-actin. **(E)** MoDCs were treated with 2-DG (7 mM) or 3-PO (25 µM) for 1h and then transfected with M8 (0.5 ng/ml). After 4h cells were infected with hCoV-229E (MOI 1) for 24h. Cells were then harvested, and expression of viral RNA was assessed by qPCR. 229E RNA expression was normalized on β-actin and results represent fold changes compared to CTRL (hCoV-229E alone), set as 100. **(F)** MoDCs were treated with 2-DG (7 mM) for 1h and then transfected with M8 (0.5 ng/ml). After 4h cells were infected with Coxsackie virus B5 (CV-B5) (MOI 1) for 24h. Cells were then harvested, and expression of viral RNA was assessed by qPCR. CV-B5 RNA expression was normalized on β-actin and results represent fold changes compared with CTRL (CV-B5 alone), set as 100.

The observation that 2-DG was sufficient to block M8 antiviral signal in MoDCs prompted us to investigate whether pharmacologic inhibition of glycolysis would interfere with M8-induced antiviral response against virus infection. To determine the importance of glycolysis in this process, MoDCs were pre-treated with 2-DG or 3-PO, transfected with M8 and subsequently infected with dengue (NGC) or the human coronavirus 229E (hCoV-229E) for an additional 24h (**Figure 5A**). M8 completely blocked dengue virus (DENV) infection, as determined by quantitative PCR (qPCR analysis) of vRNA and the inhibition of glycolysis by 3-PO reversed the antiviral effect of M8, since DENV replication was completely restored (≈110% vs. control (CTRL) (**Figure 5B**). Strikingly, 2-DG failed to reverse the antiviral effect of M8 and displayed only a partial and not significant recovery of vRNA levels (≈25%) (**Figure 5B**). As discussed below, the result may relate to an effect of 2-DG on N-linked glycosylation and virus maturation (Mondotte et al., 2007). Consistent with this hypothesis, no DENV E protein was detected in the presence of 2-DG, even in the absence of M8 treatment (**Figure 5C**). To confirm that glycolysis was required to activate the antiviral response induced by M8, a different virus model—hCoV-229E—was used to infect MoDCs (**Figure 5D**) and the inhibitory activities of 2-DG and 3-PO were determined (**Figure 5E**). As observed with DENV infection, a strong reduction in hCoV-229E vRNA expression (≈90%) was exerted by M8 and was maintained in the presence of 2-DG but not 3-PO (≈50% recovery) (**Figure 5E**). However, 2-DG reduced the levels of 229E vRNA levels even in the absence of M8, consistent with the hypothesis that block of N-glycosylation affects the infectious process of enveloped viruses, as observed also with other coronaviruses (Codo et al., 2020; Huang et al., 2021). Finally, to verify whether the antiviral effect of 2-DG on both DENV and hCoV-229E replication was related to the block of N-glycosylation, we infected MoDCs with the non-enveloped virus Coxsackie B5 (CV-B5). As expected, 2-DG did not impair CV-B5 replication and was able to strongly revert the antiviral effect elicited by M8 (**Figure 5**). In summary, the glycolytic burst triggered by RIG-I pathway stimulation in MoDCs is an essential bioenergetic requirement to trigger the antiviral activity of M8.

## Discussion

Myeloid lineage–derived DCs and macrophages are central to the host immune response to viral pathogens, and their recognition of pathogenic stimuli drives the early antiviral and inflammatory responses to pathogens. In addition to the expression of genes associated with the innate immune response, many studies have now linked changes in host metabolic reprogramming with generation of an efficient immune response. These changes serve in part to fulfill the energetic requirements of DC activation [reviewed in (Moller et al., 2022)].

Central to the innate immune response to RNA viruses are the RIG-I–like receptors that sense, bind, and respond to viral RNA in the cytosol. In the present study, we sought to examine the metabolic requirements for the induction of the interferon antiviral response following RIG-I activation using a well-characterized, sequence-optimized RIG-I agonist (M8). We demonstrate that (1) M8 induced a glycolytic switch in MoDC; (2) inhibition of glycolysis blocked the maturation of DCs, reflected in decreased surface expression of CD80, CD83, CD86, and MHC-II; (3) inhibition of glycolysis also compromised the capacity of MoDCs to induce an antiviral transcriptional program in response to M8 stimulation; and (4) inhibition of glycolysis differentially restricted the M8 antiviral response against human viruses dengue, coronavirus 229E, and Coxsackie B5.

M8 stimulation of DC induced glycolysis but not fatty acid synthesis in primary DCs (data not shown) and provided the energetic requirements to drive the RIG-I–mediated antiviral response. The use of the hexokinase inhibitor 2-DG blocked the RIG-I induction of DC activation markers CD80, CD83, and CD86 by interfering with the induction of glycolysis. In terms of RIG-I signaling to the type I IFN response, inhibition of glycolysis also blocked the activation of TBK1 kinase and consequently decreased phosphorylation of the critical transcription factors IRF3 and STAT1.

One of the genes that was impressively induced by M8 (2900-fold) was *ACOD1*, which encodes for the enzyme cis-aconitate decarboxylase, also known as immune responsive gene 1 (Irg1). Its transcription is dramatically upregulated in inflammatory conditions in response to IFN-I, and it functions as a link between Kreb’s cycle and immune response (O'Neill and Artyomov, 2019). Recently, the endogenous metabolite itaconate (ITAC), a by-product of Kreb’s cycle metabolism, has attracted considerable attention in the immunometabolism field because of its central role in modulating inflammatory and antiviral responses (Hooftman and O'Neill, 2019; Hooftman et al., 2020), as well as regulating immune tolerance and trained immunity (Dominguez-Andres et al., 2019); Irg1 is the enzyme responsible for itaconate production. Interestingly, IRG1-mediated itaconate production has been shown to limit proinflammatory cytokine and type I IFN accumulation in response to inflammatory stimuli, thus generating a negative feedback loop crucial for shaping immune responses (Mills et al., 2018; Hooftman and O'Neill, 2019; Wu et al., 2020).

Several studies have identified Irg1 as one of the most upregulated genes in response to pathogens [reviewed in (Wu et al., 2020)]; however, its role in infections is not well defined. On the one hand, Irg1 overexpression in mice has been shown to protect animals from Zika virus (ZIKV), West Nile virus (WNV), and mouse hepatitis virus (MHV) infection; conversely, other studies reported that it promotes respiratory syncytial virus (RSV) and VSV infection in mice (Wu et al., 2020). These observations suggest a dual role of Irg1 in modulating immune response against infections.

In the context of M8 stimulation of RIG-I in human MoDCs, upregulation of Irg1 coincided with a strong reduction of viral replication; the pharmacological inhibition of glycolysis by both 2-DG and 3-PO strongly blocked the IRG1 protein expression and reversed the antiviral effect elicited by M8. Despite this evidence, however, we cannot assume a direct correlation between IRG1 activity and modulation of viral replication; further investigation is necessary to understand the interrelationships between Kreb’s cycle metabolism and the role of IRG1-induced itaconate in cell- or virus-specific responses to pathogens.

An excellent previous study highlighted the idea that inhibition of glycolysis could result in different outcomes in pDC and MoDCs (Fekete et al., 2018). While production of type I interferon after stimulation of RIG-I signaling in pDC relied on oxidative phosphorylation (OXPHOS), induction of RIG-I in MoDCs resulted in glycolysis-dependent IFN production. As a consequence, pharmacological inhibition of glycolysis by 2-DG resulted in block of type I IFN release in MoDCs but not in pDC (Fekete et al., 2018).

Here, we demonstrate that inhibition of glycolysis in MoDCs impairs type I interferon signaling by disrupting the TBK1-IRF3-STAT1 axis, thereby countering the antiviral activity induced by M8. Functionally, the impaired IFN response resulted in enhanced viral replication; intriguingly, blockade of glycolytic pathway by 2-DG abrogated the antiviral effect of M8 in MoDCs, restoring Coxsackie virus replication, but failed to exert the same effect on dengue (DENV) or human coronavirus hCoV-229E; conversely, the PFK-2 inhibitor 3-PO interfered with the antiviral effects of M8 during both DENV and hCoV-229E infections. As shown in **Figures 5C, E**), 2-DG has a direct antiviral effect against DENV and 229E, as demonstrated by the absence of DENV envelope protein expression (**Figure 5C**) and the significantly reduced levels of 229E RNA (**Figure 5E**). We hypothesized that the repressive role of 2-DG in N-linked glycosylation could explain these observations. In fact, 2-DG is structurally similar to mannose and competes for N-linked glycosylation of proteins, a critical process for viral envelope protein stability and secretion (Berthe et al., 2018). Differently from mannose, in the presence of 2-DG, the intermediate precursors of lipid-linked oligosaccharides cannot be further extended, resulting in the interruption of N-glycosylation process. For viral envelope, this leads to an incomplete protein maturation and to the block of secretion of newly synthesized viral particles. As a consequence, the addition of 2-DG blocked the N-linked glycosylation of DENV E protein and impaired viral propagation (Mondotte et al., 2007) (Courageot et al., 2000); a similar effect was observed during hCoV-229E infection (Huang et al., 2021). To confirm our hypothesis that 2-DG exerts a direct antiviral effect against enveloped viruses, we infected MoDCs with the non-enveloped virus Coxsackie B5. Results obtained with CV-B5 indicate that the antiviral activity of 2-DG is restricted to enveloped viruses, and we further confirm that blocking glycolysis and, subsequently, the antiviral response elicited by M8, results in increased viral replication. In conclusion, our findings characterize the glycolytic pathway as an essential component in the orchestration and fine tuning of the type I IFN antiviral response in MoDCs, with implications for the development of therapeutic options in the context of viral infection.

## Methods

### Monocyte Isolation and Differentiation Into MoDCs

Istituto Superiore di Sanità Review Board approved the use of blood from healthy donors (AOO-ISS - 14/06/2020 - 0020932). A written informed consent was obtained from all subjects involved, and procedures were completed according to the European Union guidelines and the Declaration of Helsinki. Peripheral blood mononuclear cells (PBMCs) were isolated from buffy coats by Ficoll Paque Plus (GE Healthcare, Chicago, IL, USA) following the manufacturer’s instructions. For DC differentiation, monocytes were isolated from fresh PBMCs by magnetic selection using CD14 MicroBeads (Miltenyi Biotec) and a magnetic cells separator as per kit instructions (Miltenyi Biotec). Purified CD14+ monocytes were seeded at a density of 1 × 10^6^ cells/ml in and cultured in RPMI supplemented with 10% certified fetal bovine serum (FBS) (Thermo Fisher Scientific), 1% of penicillin-streptomycin solution (P/S, 10,000 U/ml penicillin and 10 mg/ml streptomycin sulfate, Euroclone), 50 ng/ml of GM-CSF (Granulocyte-macrophage colony-stimulating factor) (Miltenyi Biotec) and 25 ng/ml of IL-4 (Miltenyi Biotec) at 37°C (5% CO2). After 5 days, the phenotype of these cells was concordant with an immature DC population: DC-SIGN^high^, CD1a^high^, and CD14^very low^. For M8 transfection experiments, MoDCs were seeded at 10^6^ cells/ml in 12-well plates and incubated at 37°C. The right volume of M8 for the desired final concentration was mixed with 5 μl of HiPerFect (Qiagen) and 100 µl of Opti-Mem (Thermo Fisher Scientific) for 10 min at room temperature before the mixes were added to the cells in culture.

### M8 Generation

M8 was synthesized using Megascript T7 Transcription Kit (Thermo Fisher Scientific) with synthetic oligonucleotides (Eurofins Genomics) and following the manufacturer’s instructions. Templates used were

Fw: GAA ATT AAT ACG ACT CAC TAT AGA CGA AGA CCA CAA AAC CAG ATA AAA AAA AAA AAA AAA AAA AAAAAA ATA ATT TTT TTT TTT TTT TTT TTT TTT TTT ATCTGG TTT TGT GGT CTT CGT C

Rev: GAC GAA GAC CAC AAA ACC AGA TAA AAA AAA AAA AAA AAA AAA AAA AAA TTA TTT TTT TTT TTT TTT TTT TTT TTT TTA TCT GGT TTT GTG GTC TTC GTC TAT AGT GAG TCG TAT TAA TTT C

Synthesized RNA was then purified using Nucleospin MiRNA Kit (Macherey-Nagel), and its concentration was assessed using Nanodrop 2000 (Thermo Fisher Scientific).

### Virus Production, Quantification, and Infection

DENV production was performed as previously described (Ferrari et al., 2020). Briefly, C6/36 cells were infected with DENV-2 NGC at low multiplicity of infection (MOI) (0.05); after 7 days, the supernatant of infected cells was collected and cleared by centrifugation. The virus was then concentrated and purified by ultracentrifugation on a 20% sucrose cushion. Viral titer was determined by flow cytometry analysis of DENV E protein expression in Vero E6–infected cells (Lambeth et al., 2005). Titers were expressed as IU/ml. In infection experiments, MoDCs were infected in a small volume of medium without FBS for 1h at 37°C and then incubated with complete medium for 24h prior to analysis.

hCoV-229E was obtained from American Type Culture Collection (ATCC) (ATCC VR-740) was grown and maintained in Huh-7 cells. MoDCs were seeded in six-well plates and infected with hCoV-229E in 500 µl of medium without FBS for 1h at 37°C and then incubated with complete medium for the indicated time points prior to analysis.

Coxsackie virus—strain B5 (CV-B5) was a kind gift from Professor Ombretta Turriziani (Sapienza University, Rome, Italy) and was grown and maintained in Vero E6 cells. MoDCs were seeded in six-well plates and infected with CV-B5 in 500 µl of medium without FBS for 1h at 37°C and then incubated with complete medium for the indicated time points prior to analysis.

### qPCR

RNA was isolated by column separation using the RNeasy Kit (Qiagen) following the manufacturer’s instructions, and the concentration was measured with Nanodrop 2000. A quantity of RNA in the range 200–500 ng was used for cDNA synthesis using the PrimeScript RT-PCR Kit (Takara-Bio). qPCR was then performed using Taqman Fast Advanced MasterMix with Universal ProbeLibrary Probes (Roche) with specific primers designed using the Roche Lifescience Assay Design Center (https://lifescience.roche.com/en_it/brands/universal-probe-library.html#assay-design-center) on a StepOnePlus Real-Time PCR System (Thermo Fisher Scientific). A relative quantification method was used, with TATA-Box Binding Protein (TBP) or β-actin as housekeeping genes.

### Protein Extraction and Immunoblot Analysis

MoDCs were washed in ice-cold phosphate-buffered saline (PBS) and lysed in lysis buffer [150 mM NaCl, 50 mM Tris-HCl (pH 7.6), 5 mM EDTA (pH 8), 1% Nonidet-P-40 (NP-40), 0,5% sodium deoxycholate, 0.1% sodium dodecyl sulfate (SDS)], in the presence of Halt™ Protease and Phosphatase Inhibitor Cocktail (Thermo Fisher Scientific), and benzonase (Sigma-Aldrich) for 25 min on ice. Protein concentration was determined using the Pierce bicinchoninic (BCA) protein assay kit (Thermo Fisher Scientific). Proteins were resolved by SDS–polyacrylamide gel electrophoresis on 4%–20% precast Novex Tris-Glycine gradient gels (Thermo Fisher Scientific) and blotted onto polyvinylidene difluoride (PVDF) membranes (GE Healthcare). Blots were incubated with the indicated primary antibodies, at 1:1000 dilution in 5% (w/v) bovine serum albumin, overnight at 4°C, extensively washed with TBS-T and after incubation with horseradish peroxidase (HRP)–labeled goat anti-rabbit or goat anti-mouse Abs (Cell Signaling Technology), developed with the enhanced chemiluminescence (ECL) detection system as per manufacturer’s instructions (Cyanagen). Primary antibodies anti–phospho-IRF3 at Ser^396^, anti-IRF3, anti phosphoSTAT1 at Tyr^701^, anti-STAT1, anti–phospho-IkBa at Ser^32^, anti-IkBa, anti–phospho-TBK1, anti IRG1, and anti–β-actin were all purchased from Cell Signaling.

### Flow Cytometry

Staining for cell surface markers was performed using standard incubation protocols and staining with anti–HLA-DR-APC (clone LT-DR) (Immunotools), anti–CD86-Brilliant Violet 421 (clone IT2.2) (BioLegend), anti–CD83-FITC (clone HB15e), anti–CD80-PE (clone L307.4) (both from BD Biosciences) antibodies. All the experiments were performed on BD FacsCanto II (BD Biosciences).

### Magnetic Luminex Assay

Supernatants of M8-transfected MoDCs were analyzed on a 96-well plate for the presence of the following cytokines: IFN-β, IFN-γ, IL-6, and TNF-α. The fluorescence responses and concentrations of cytokines were obtained using a human premixed Multi-Analyte kit (R&D systems) and analyzed with a MAGPIX system and the accompanying xPONENT Software. All reagents were provided with the kit and were prepared according to the manufacturer’s recommendations; reconstituted standards were serially diluted 1:3 in calibrator diluent, which was used as background. A microparticle cocktail, biotin-antibody cocktail, and streptavidin-PE were diluted in their specific buffers immediately before the assay. The Luminex protocol was followed exactly; 50 μl of sample and standard were incubated for 2h with 50 μl of microplate cocktail, then the plate was washed and 50 μl of biotin-antibody cocktail was added. After 1h incubation, the plate was washed and 50 μl of streptavidin-PE was added for 30 min, followed by a final wash and resuspension in 100 μl of wash buffer. All incubations were performed at room temperature on a microplate shaker at 800 rpm. Both standards and samples were tested in duplicate. The concentration values and detection limits were determined from standard curves generated from each kit’s standards using the weighted 5PL curve fitting procedure. To maximize the number of concentration values available for analysis, the extrapolated values were included.

### Lactate Production

The concentration of lactate in supernatant of M8-treated MoDCs was measured using a luminometric Lactate-Glo assay kit (Promega), according to the manufacturer’s protocol. The luminometry was measured with GloMax^®^-Multi Detection System microplate reader (Promega). Results were quantified using a standard curve.

### Glucose Uptake

To determine the rate of cellular glucose uptake in MoDCs, cells were treated with M8 for 18h, then washed with PBS and subjected to glucose deprivation for 40 min. Glucose uptake was initiated by addition of 1 mM 2-DG for 20 min, before cells were lysed and intracellular glucose was measured using a Glucose Uptake Colorimetric Assay Kit (BioVision) and an iMark Microplate Absorbance Reader (Bio-Rad).

### Seahorse XF Analyzer Respiratory Assay

Cellular OCR and ECAR were detected using XF Cell Mito Stress Test (Agilent) measured by the extracellular flux analyzer XFe96 (Agilent Technologies, Santa Clara, CA, United States). Briefly, 50,000 MoDCs per well in Poly-D-Lysine–coated Seahorse XFe96 microplates were cultured in RPMI complete media supplemented with 10% (v/v) heat-inactivated FBS for 24h. The cells were either transfected with HiPerFect only (control) or treated with M8 (50 ng/ml) for 24h. At the end of treatment, the cells were switched to 180 μl of XF base medium (non-buffered Dulbecco's Modified Eagle Medium (DMEM) with no phenol red supplemented with 10 mM glucose, 10 mM pyruvate, and 2 mM L-glutamine at pH 7.4), washed twice and incubated for 30 min in a CO2-free incubator at 37°C. MoDCs were obtained by 6 days cultured CD14+ human monocytes (in RPMI supplemented with 10% certified FBS (Thermo Fisher Scientific), 1% of penicillin-streptomycin solution (P/S, 10,000 U/ml penicillin and 10 mg/ml streptomycin sulfate, Euroclone), 50 ng/ml of GMCSF (Miltenyi Biotec) and 25 ng/ml of IL-4 (Miltenyi Biotec) at 37°C (5% CO2). The sensor cartridge for XFe analyzer was hydrated in a 37 °C non-CO_2_ incubator a day before the experiment. According to the manufacturer’s instructions, stressors concentrations were optimized and added as follows: 1 μM oligomycin as complex V inhibitor, 1 μM FCCP (uncoupler agent), and 0.5 μM rotenone/antimycin A (inhibitors of complex I and complex III). OCR was normalized for total protein/well/50,000 cells. Each sample/treatment was analyzed in at least 10 wells for experiment, two independent experiments were carried out; the figure represents one sample experiment. Statistical analysis has been performed on each experiment by using one way analysis of variance (ANOVA) followed by Bonferroni *post-hoc* comparison test. *P* < 0.05 has been considered significant.

## Data Availability Statement

The original contributions presented in the study are included in the article/supplementary material. Further inquiries can be directed to the corresponding author.

## Ethics Statement

The studies involving human participants were reviewed and approved by Istituto Superiore di Sanità Review Board (AOO-ISS - 14/06/2020 - 0020932). The patients/participants provided their written informed consent to participate in this study.

## Author Contributions

AZ and EP equally contributed to the conception, experimentation, and preparation of the manuscript and figures. DDC, MA, and SR participated in the experimentation and preparation of the manuscript, including text and figures. AP, FC, ME, EC, and DO provided supervision, contributing to writing and editing the manuscript. JH provided supervision, wrote and edited the manuscript. All authors have read and agreed to the published version of the manuscript.

## Funding

This project was supported by funding from the Italian Association for Cancer Research (IG-2019-22891) and from the European Union’s Horizon 2020 research and innovation programme under grant agreement no. 813343 for the Marie Curie ITN INITIATE program (to JH). Funding from Sapienza University of Rome for the HypACB platform for Seahorse experiments is also gratefully acknowledged (grant GA116154C8A94E3D to FC).

## Conflict of Interest

The authors declare that the research was conducted in the absence of any commercial or financial relationships that could be construed as a potential conflict of interest.

## Publisher’s Note

All claims expressed in this article are solely those of the authors and do not necessarily represent those of their affiliated organizations, or those of the publisher, the editors and the reviewers. Any product that may be evaluated in this article, or claim that may be made by its manufacturer, is not guaranteed or endorsed by the publisher.

## Glossary

**Table:**

ACL	ATP-citrate lyase
ADE	antibody-dependent enhancement
ARE	antioxidant response element
CAT	catalase
cGAS	cyclic GMP-AMPsynthase
CHOP	C/EBP-Homologous Protein
CLEC5A	C-type lectin domain family 5 member A
DENV	dengue virus
DF	dengue fever
DHS	DENVinduced hemorrhagic fever
DMSO	dimethyl sulfoxide
DSS	DENV-induced shock syndrome
EIF2A	eukaryotic translation initiation factor 2A
FAS	fatty acid synthase
G6PD	glucose-6-phosphate dehydrogenase
GAPDH	glyceraldehyde-3-phosphate dehydrogenase
GCLC	glutamate–cysteine ligase catalytic
GCLM	glutamate–cysteine modifier
GFP	green fluorescent protein
GPX2	glutathione peroxidase 2
GSH	glutathione
HO-1	heme oxygenase 1
Hpi	hours postinfection
IFIT1	interferon-induced protein with tetratricopeptide repeats 1
IFN	interferon
IkBa	nuclear factor of k light polypeptide gene enhancer in B cells inhibitor a
IL-1b	interleukin-1b
IL-6	interleukin-6
IL-8	interleukin-8
IRF3	interferon regulatory factor 3
IRG1 IFN	regulated gene 1 or ACOD1 cisaconitate decarboxylase
Keap1	kelch-like ECH-associated protein 1
KO	knockout
MDA	malondialdehyde
MDA5	melanoma differentiation-associated protein 5
ME1	malic enzyme 1
NADP+-dependent	cytosolic
MMP-9	matrix metalloproteinase 9
MoDCs	monocyte-derived dendritic cells
NF-kB	nuclear factor k-light-chain-enhancer of activated B cells
NOX	NADPH-oxidase
Nrf2	nuclear factor erythroid 2–related factor 2
NS1	non-structural protein 1
PARP	poly(ADP-Ribose) polymerase
PERK	protein kinase R-like ER kinase
PGD	6-phosphogluconate dehydrogenase
PPP	pentose phosphate pathway
PUMA P53	upregulated modulator of apoptosis
RIG-I	retinoic acid-inducible gene I
ROS	reactive oxygen species
SCD1	stearoyl CoA desaturase
SFN	sulforaphane
SLC7A11	solute carrier family 7 member 11 (cystine/glutamate transporter)
SOD	superoxide dismutase
STAT1	signal transducer and activator of transcription 1
STING	stimulator of interferon genes
TKT	transketolaseisoform 1
TNFa	tumor necrosis factor alpha
TRAIL	TNF-related apoptosis inducing ligand
